# Circ_0067680 expedites the osteogenic differentiation of human bone marrow-derived mesenchymal stem cells through miR-4429/CTNNB1/Wnt/β-catenin pathway

**DOI:** 10.1186/s13062-021-00302-w

**Published:** 2021-10-14

**Authors:** Yuansheng Huang, Su Wan, Min Yang

**Affiliations:** 1 Traumatic Orthopedics, Yijishan Hospital of Wannan Medical College, Wuhu, 241001 Anhui China; 2 Department of Gynecology, Yijishan Hospital of Wannan Medical College, No. 2, Zheshan West Road, Wuhu, 241001 Anhui China

**Keywords:** Osteogenic differentiation, circ_0067680, miR-4429, CTNNB1, Wnt/β-catenin signaling pathway

## Abstract

**Background:**

Human bone marrow-derived mesenchymal stem cells (hBMSCs) are the primary source of osteoblasts in vivo. Emerging literatures have unveiled that circular RNAs (circRNAs) are actively drawn in the osteogenic differentiation of mesenchymal stem cells (MSCs). This research mainly illuminated the role of circ_0067680 as well as its regulatory mechanism in osteoblastic differentiation.

**Methods:**

In this study, RT-qPCR was to measure the expression of circ_0067680. Functional assays were implemented to assess the role of circ_0067680 in osteogenic differentiation. Besides, RNA pull down, RIP and luciferase reporter assays were carried out to investigate the regulatory mechanism of circ_0067680.

**Results:**

Circ_0067680, which derived from its host gene divergent protein kinase domain 2A (C3orf58), was up-regulated during osteogenic differentiation of hBMSCs. Besides, circ_0067680 deficiency impeded the osteoblastic differentiation of hBMSCs. Moreover, circ_0067680 served as a ceRNA via sequestering miR-4429 to regulate the expression of catenin beta 1 (CTNNB1), thereby activating the Wnt/β-catenin signaling pathway.

**Conclusion:**

Circ_0067680 accelerated hBMSCs osteogenic differentiation by the miR-4429/CTNNB1/Wnt/β-catenin signaling, which might be used as a potential biomarker for osteoblastic differentiation.

**Graphic abstract:**

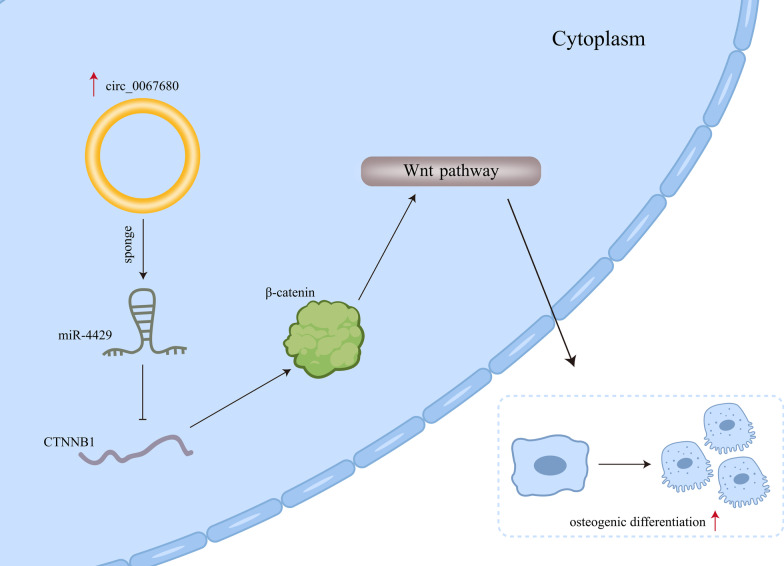

**Supplementary Information:**

The online version contains supplementary material available at 10.1186/s13062-021-00302-w.

## Background

As reported previously, the occurrence rate of fractures among the elderly constantly increases due to bone loss with age [1]. Human bone marrow-derived mesenchymal stem cells (hBMSCs) are multipotent cells for their potential of multipotential differentiation, self-renewal, immune regulation and so on [2]. Therefore hBMSCs become important regulators in bone formation and differentiation [3]. Hence, exploring the mechanism underlying the osteogenic differentiation of hBMSCs has a significant meaning in the bone formation and bone maintenance [4].

Circular RNAs (circRNAs) are capable to exert important functions in osteoblastic differentiation [5]. For instance, Guo et al. have demonstrated that circRNA-23525 accelerates the osteogenic differentiation of adipose-derived mesenchymal stem cells through serving as a sponge for miR-30a-3p [6]. Han et al. have revealed that circ_0076690 influences the osteogenic differentiation of hBMSCs via interacting with miR-152 [7]. Li et al. have validated the CDR1as/miR-7/GDF5 axis in the osteoblastic differentiation of PDLSCs [8]. Nevertheless, the roles of most circRNAs in the development of osteoblastic differentiation remain unclear. C3orf58 has been reported to be correlated to with the cell-cycle progression of cardiomyocytes. Besides, C3orf58 has been determined to be up-regulated in the differentiated human adipose-derived stem cells. Therefore, we speculated that circ_0067680 whose host gene was C3orf58 might also influence the process of osteogenic differentiation.

The Wnt/β-catenin pathway, also named as the canonical Wnt pathway, is strongly linked with bone formation [9]. Abnormal expression of CTNNB1, which can encode β-catenin, is a key gene of the Wnt signaling and is strongly related to the development of diseases, osteogenic differentiation included [10]. Targeting the Wnt/β-catenin pathway, especially CTNNB1, might be a novel method for osteoporosis treatment.

In this research, we concentrated on uncovering the potential of a novel circRNA circ_0067680, which derived from its host gene divergent protein kinase domain 2A (C3orf58) in osteogenic differentiation of hBMSCs. Besides, the relation between circ_0067680 and the Wnt/β-catenin pathway was investigated as well.

## Results

### Circ_0067680 expression is high in the differentiation of hBMSCs into osteoblasts

**Fig. 1:**
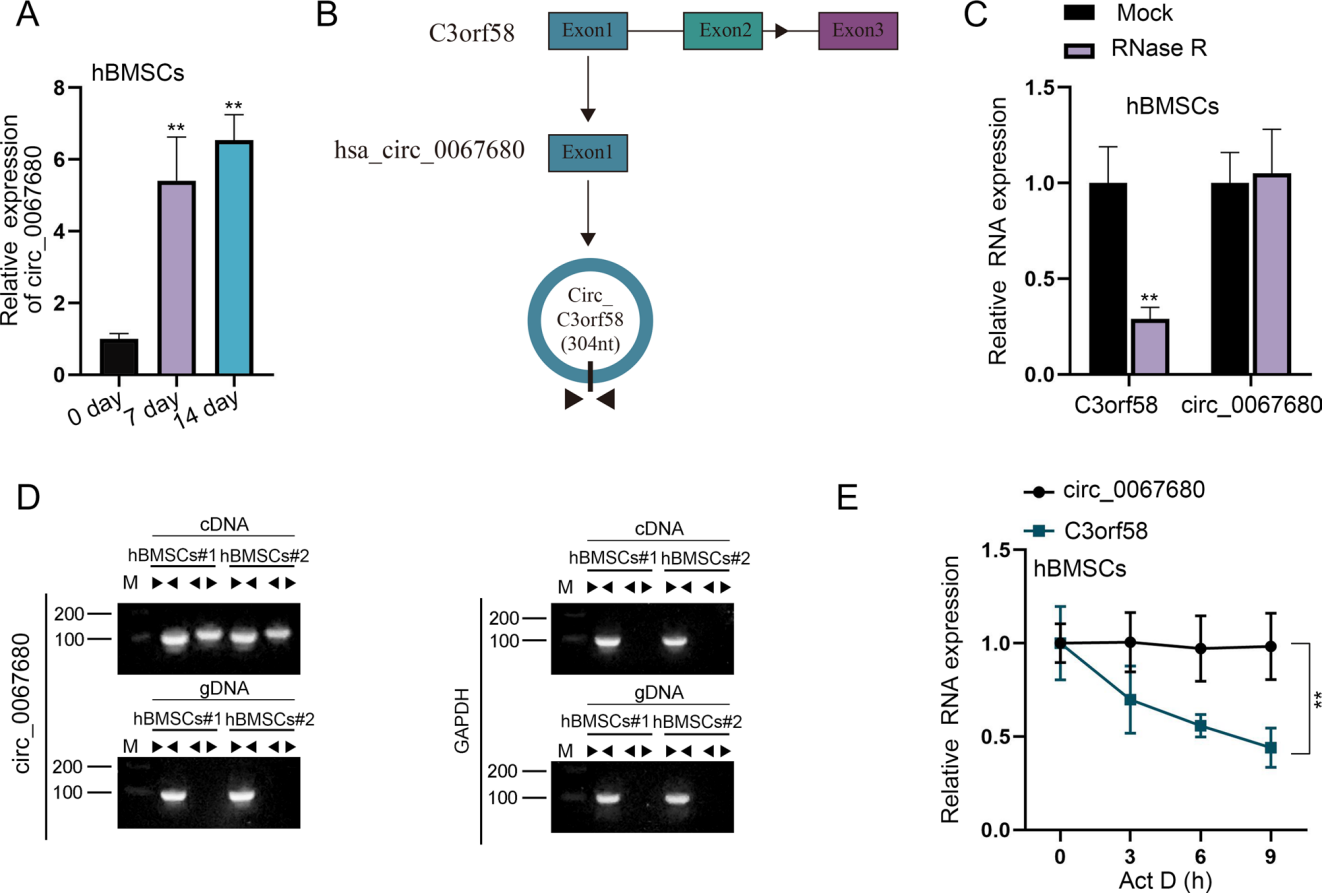
Circ_0067680 expression is high in the differentiation of hBMSCs into osteoblasts. **A** RT-qPCR examined circ_0067680 expression in hBMSCs after induction of osteogenic differentiation at 7 and 14 days. **B** Sequence diagram of cyclization site analysis of circ_0067680. **C** RT-qPCR analysis of the expression of circ_0067680 and linear-C3orf58 in hBMSCs treated without or with RNase R. **D** Agarose gel electrophoresis confirmed the existence of circ_0067680. **E** The stability of circ_0067680 and linear-C3orf58 was detected after adding Act D. **P < 0.01

To induce osteogenic differentiation, osteogenic medium was applied using α-MEM added with 1% antibiotics, 10% FBS, 0.2 mM ascorbic acid, 10 mM β-glycerophosphate and 100 nM dexamethasone. We used CCK-8 to assess the viability in hBMSCs after induction of osteogenic differentiation at 7 and 14 days. As shown in Additional file 1: Fig. S1A, the cell viability was increased in a time-dependent way. Besides, ALP staining manifested that the ALP activity was reduced with the time increased (Additional file 1: Fig. S1B). Consistently, the expression of osteogenic-linked genes (Runx2, OSX and OCN) was significantly up-regulated as time goes (Additional file 1: Fig. S1C–S1D). Through RT-qPCR analysis, we discovered that circ_0067680 was up-regulated during the osteogenic differentiation of hBMSCs (0, 7 and 14 days) (Fig. 1A). As demonstrated in Fig. 1B, the genomic location of circ_0067680 was predicted. RT-qPCR confirmed that after adding RNase R, linear-C3orf58 level was digested while circ_0067680 was counteractive to RNase R degradation (Fig. 1C). Additionally, the existence of circ_0067680 was verified by the finding that circ_0067680 could only be amplified with divergent primers from cDNA instead of genomic DNA (gDNA) whereas convergent primers could amplify linear-C3orf58 with both cDNA and gDNA (Fig. 1D). At the same time, after adding Actinomycin D (Act D), we observed that circ_0067680 was more stable than linear-C3orf58 (Fig. 1E).

### Circ_0067680 knockdown hinders the proliferation and osteogenic differentiation of hBMSCs

**Fig. 2:**
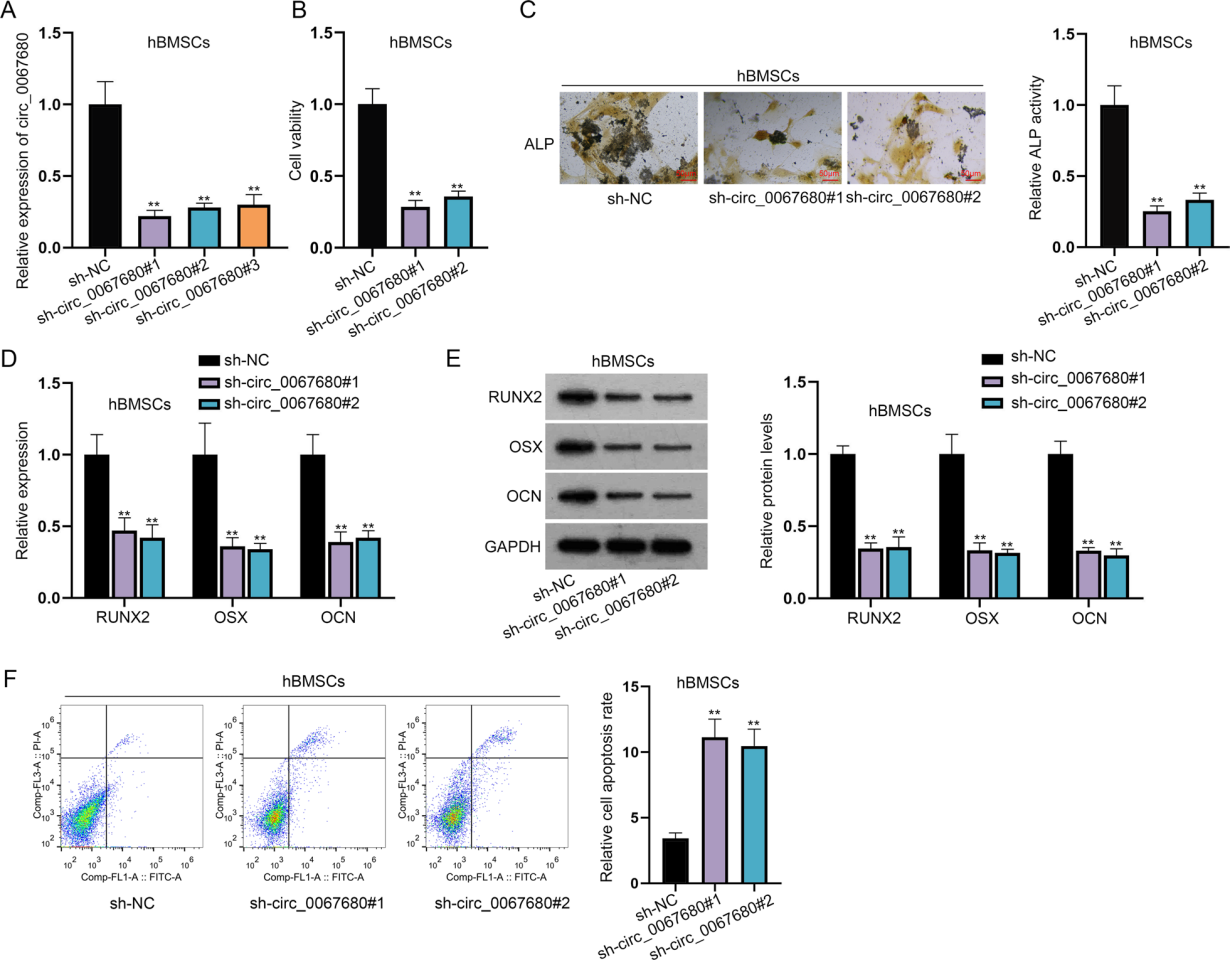
Circ_0067680 knockdown hinders the osteogenic differentiation of hBMSCs. **A** Circ_0067680 was knocked down in hBMSCs and was verified by RT-qPCR. **B** CCK-8 assay detected the cell viability in hBMSCs after circ_0067680 silence. **C** ALP activity from ALP staining assay when circ_0067680 was down-regulated. **D**, **E** The expression of RUNX2, OSX and OCN was analyzed by RT-qPCR and western blot analyses after circ_0067680 was silenced. **F** Flow cytometry was used to detect the condition of cell apoptosis when transfected with sh-circ_0067680#1/2. **P < 0.01

To assess circ_0067680 function on hBMSCs proliferation and osteogenic differentiation, circ_0067680 was knocked down in hBMSCs and the inhibition efficiency was certified by RT-qPCR. Sh-circ_0067680#1 and #2 were chosen for further study because of higher interference efficiency (Fig. 2A). Then, CCK-8 results suggested that silencing circ_0067680 inhibited cell viability (Fig. 2B). ALP staining disclosed that when circ_0067680 was down-regulated, ALP activity was significantly reduced (Fig. 2C). Moreover, depletion of circ_0067680 led to the decrease on the expression of RUNX2, OSX and OCN which were markers of osteogenic differentiation (Fig. 2D, E). To confirm the condition of the cells transfected with sh-circ_0067680#1/2, we used the flow cytometry analysis to analyze the cell apoptosis. Cell apoptosis rate enhanced when circ_0067680 was interfered (Fig. 2F).

### Circ_0067680 interacts with miR-4429

**Fig. 3:**
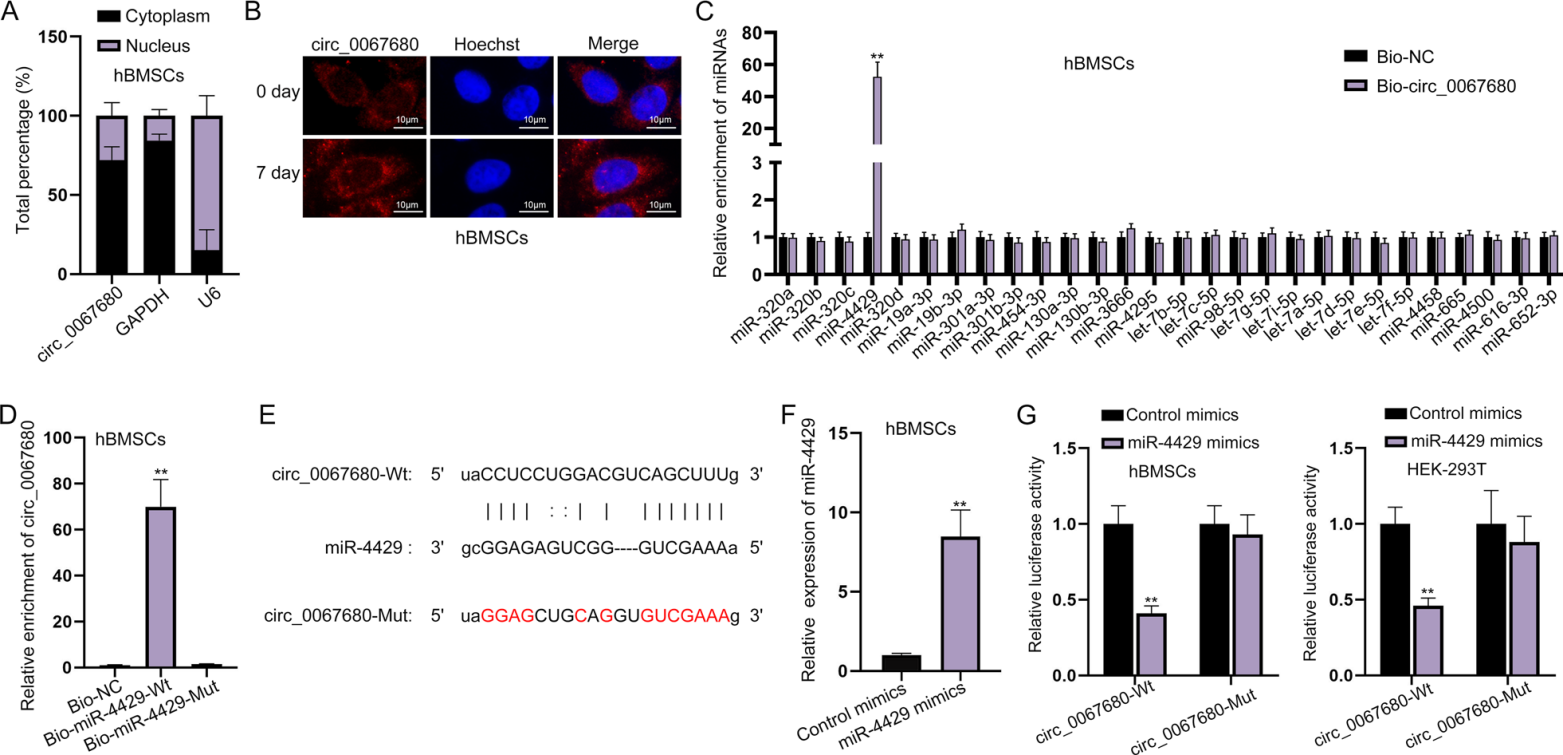
Circ_0067680 interacts with miR-4429. **A**, **B** Subcellular fractionation and FISH assays detected the distribution of circ_0067680 in hBMSCs. **C** RNA pull down assay validated the interaction between circ_0067680 and miRNAs. **D** RNA pull down assay validated the interaction between circ_0067680 and miR-4429. **E** The binding sequence between circ_0067680 and miR-4429. **F** Transfection of miR-4429 mimics in hBMSCs was verified by RT-qPCR. **G** Luciferase reporter assay verified the binding between circ_0067680 and miR-4429. **P < 0.01

Recent studies have demonstrated that cirRNAs exert functions in the development of diseases via regulating their host genes [11]. Thus, we assumed that circ_0067680 might promote hBMSCs proliferation and osteogenic differentiation by modulating its host gene C3orf58. However, we also observed that when circ_0067680 was silenced, the expression of C3orf58 exhibited no obvious change (Additional file 2: Fig. S2A–B), which excluded our conjecture. To further probe the regulatory mechanism of circ_0067680 in hBMSCs, we detected the distribution of circ_0067680 in hBMSCs with the application of subcellular fractionation and FISH assays. The result indicated that circ_0067680 was mainly presented in the cytoplasm of hBMSCs (Fig. 3A, B). As we all know, cytoplasmic circRNA can interact with specific miRNAs to exert functions. Therefore, we used starBase (http://starbase.sysu.edu.cn/index.php) to predict the miRNAs bound to circ_0067680. Based on the condition of CLIP Data ≥ 5, we discovered 28 possible miRNAs. Through RNA pull down experiment, we discovered that only miR-4429 significantly interacted with circ_0067680 in hBMSCs (Fig. 3C). Hence, miR-4429 was chosen for subsequent study. Besides, RNA pull down assay also displayed the strong affinity of circ_0067680 and miR-4429 (Fig. 3D). The binding sequence between circ_0067680 and miR-4429 was exhibited in Fig. 3E. Before the implementation of luciferase reporter assay, miR-4429 was overexpressed after transfection of miR-4429 mimics (Fig. 3F). Furthermore, the binding between circ_0067680 and miR-4429 was verified by luciferase reporter assay (Fig. 3G), as shown that miR-4429 elevation obviously weakened the luciferase activity of circ_0067680-Wt, while barely influenced that of circ_0067680-Mut.

### Circ_0067680 activates the Wnt/β-catenin signaling pathway

**Fig. 4:**
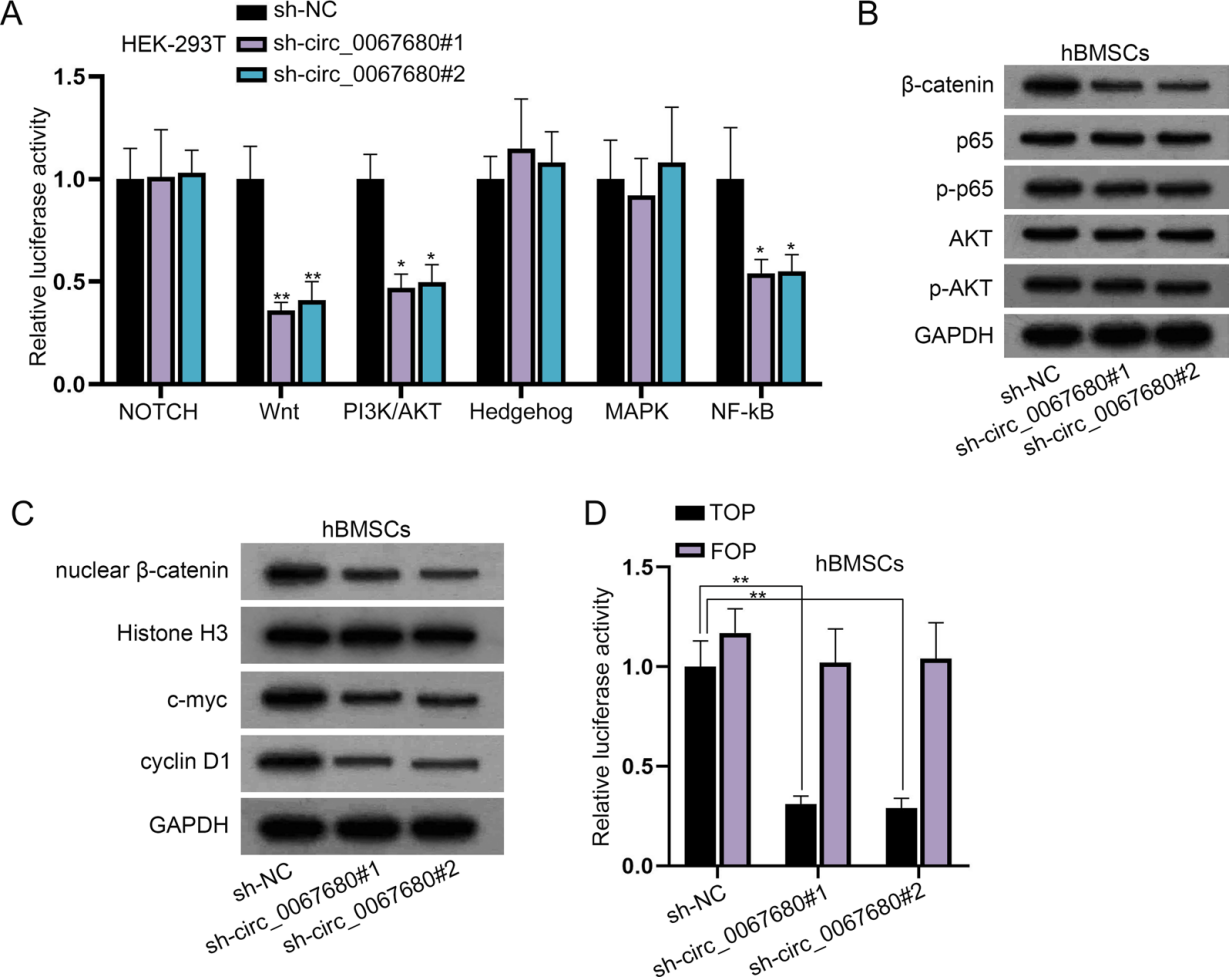
Circ_0067680 participates in the activation of Wnt/β-catenin signaling pathway. **A** The activity of common signaling pathways when circ_0067680 was depleted was detected by luciferase reporter assay. **B** Protein levels of β-catenin, p65, p-p65, AKT and p-AKT were analyzed under the condition of circ_0067680 inhibition. **C** The expression of nuclear β-catenin, c-myc, cyclin D1 at protein level was detected when circ_0067680 was inhibited. **D** TOP/FOP flash assay assessed the activity of Wnt/β-catenin signaling pathway after circ_0067680 was silenced. *P < 0.05, **P < 0.01

By measuring the activity of downstream transcription factors, the activity of signal transduction pathway can be detected quickly and sensitively. It has been reported that signaling pathway can be involved in osteogenic differentiation [12–15], we wanted to explore whether circ_0067680 could affect osteogenic differentiation by regulating downstream signaling pathways. Therefore, we detected the luciferase activity of these pathways by using the signaling pathway kit (Cignal Finder Reporter Array, QIAGEN, Dusseldorf, Germany). Based on luciferase reporter assay, we noticed that circ_0067680 silence reduced the luciferase activity of Wnt signaling pathway, PI3K/AKT signaling pathway and NF-kB signaling pathway (Fig. 4A). Western blot further analyzed that circ_0067680 interference lessened β-catenin level but barely affected the levels of p-p65 and p-AKT, which indicated that circ_0067680 was correlated with Wnt signaling pathway (Fig. 4B). The protein levels of related factors in the Wnt/β-catenin signaling pathway (nuclear β-catenin, c-myc, cyclin D1) were also reduced due to circ_0067680 depletion (Fig. 4C). TOP/FOP flash luciferase reporter assay proved that circ_0067680 depletion decreased the activity of Wnt signaling pathway (Fig. 4D).

### CTNNB1 is targeted by miR-4429

**Fig. 5:**
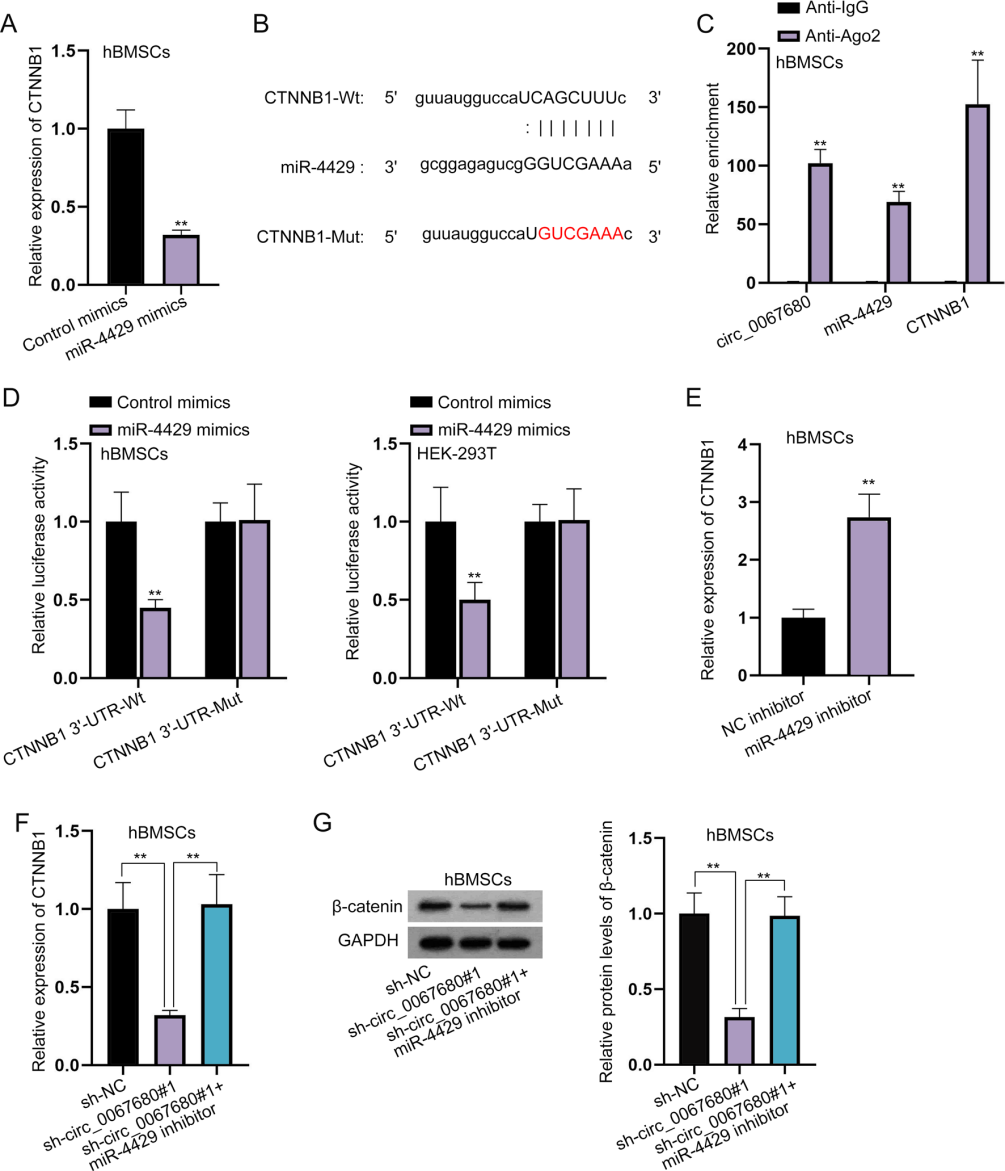
CTNNB1 is targeted by miR-4429. **A** CTNNB1 expression was examined after transfection of miR-4429 mimics in hBMSCs by RT-qPCR. **B** The binding regions between miR-4429 and CTNNB1. **C** RIP assay displayed the abundance of circ_0067680, miR-4429 and CTNNB1 in Ago2 antibody. **D** The binding between miR-4429 and CTNNB1 was verified by luciferase reporter assay. **E** Transfection of miR-4429 inhibitor to reduce miR-4429 expression. **F**, **G** The expression of CTNNB1 was examined in the sh-NC group, sh-circ_0067680#1 group and sh-circc_0067680#1 + miR-4429 inhibitor group. **P < 0.01

Intriguingly, we discovered that CTNNB1 which encoded β-catenin was a target gene of miR-4429. Thereafter we speculated that circ_0067680 might regulate CTNNB1 expression to activate the Wnt/β-catenin signaling pathway. RT-qPCR analyzed that after miR-4429 was up-regulated, CTNNB1 expression was decreased (Fig. 5A). The binding sites between miR-4429 and CTNNB1 were demonstrated in Fig. 5B. The interaction among circ_0067680, miR-4429 and CTNNB1 was also displayed by RIP assay (Fig. 5C). Luciferase reporter assay uncovered that miR-4429 mimics dramatically declined the luciferase activity in the CTNNB1 3’-UTR-Wt group (Fig. 5D). The expression of CTNNB1 was improved after transfection of miR-4429 inhibitor, which indicated the interference efficiency of miR-4429 inhibitor (Fig. 5E). Besides, the decreased CTNNB1 expression caused by circ_0067680 deficiency was completely restored by down-regulation of miR-4429 (Fig. 5F, G).

### Circ_0067680 contributes to the progression of osteogenic differentiation of hBMSCs via targeting miR-4429/CTNNB1 axis

**Fig. 6:**
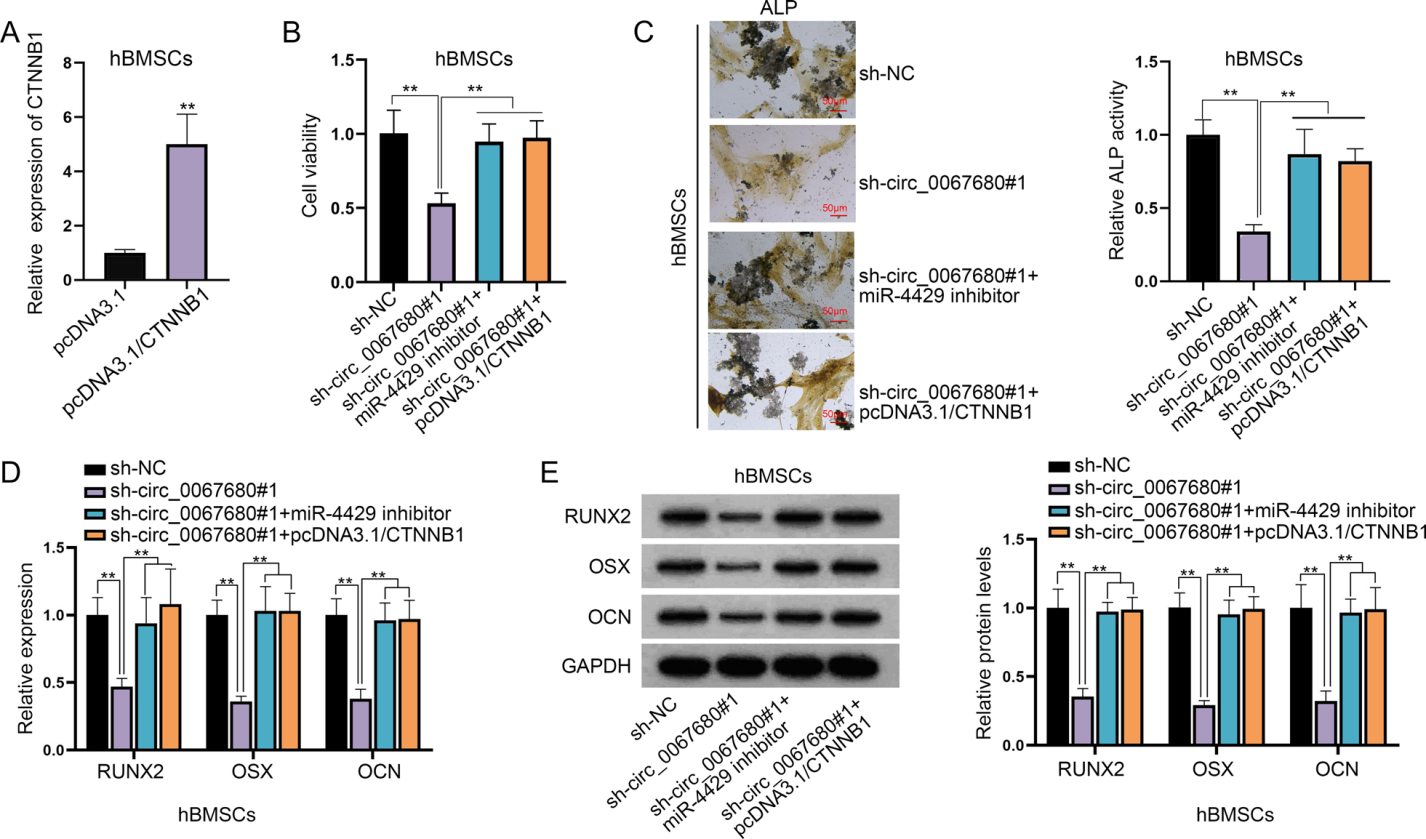
Circ_0067680 contributes to the progression of osteogenic differentiation of hBMSCs via targeting miR-4429/CTNNB1 axis. **A** CTNNB1 expression was elevated in hBMSCs and was verified by RT-qPCR. **B** CCK-8 assay exmained hBMSCs viability. **C** ALP accumulation was observed in hBMSCs transfected with sh-NC, sh-circ_0067680#1, sh_circ_0067680#1 + miR-4429 inhibitor and sh-circ_0067680#1 + pcDNA3.1/CTNNB1 plasmids. **D**, **E** Expression of RUNX2, OSX and OCN was tested in the sh-NC group, sh-circ_0067680#1 group, sh-circ_0067680#1 + miR-4429 inhibitor group and sh-circ_0067680#1 + pcDNA3.1/CTNNB1 group. **P < 0.01

Before the implementation of rescue assays, CTNNB1 expression was enhanced in hBMSCs (Fig. 6A). From the experimental results of CCK-8 assay, we found that the reduced cell viability caused by circ_0067680 silence was offset upon co-transfection of miR-4429 inhibitor or pcDNA3.1/CTNNB1 (Fig. 6B). Through ALP staining, we found that circ_0067680 knockdown inhibited ALP accumulation while this effect was completely rescued by miR-4429 inhibition or CTNNB1 overexpression together (Fig. 6C). Similarly, the decrease on the protein levels of RUNX2, OSX and OCN on account of circ_0067680 down-regulation was completely offset by simultaneous miR-4429 inhibition or CTNNB1 up-regulation (Fig. 6D, E).

## Discussion

Mounting evidence has demonstrated that the investigation on the functions of circRNAs provide new avenues for understanding the osteogenic differentiation and provide novel insights for the diagnosis and treatment of bone diseases [16]. C3orf58 has been reported to be correlated with the cell-cycle progression of cardiomyocytes [17]. Besides, C3orf58 has been determined to be up-regulated in the differentiated human adipose-derived stem cells [18]. Therefore, we speculated that circ_0067680 whose host gene was C3orf58 might also influence the process of osteogenic differentiation. In our study, we observed that circ_0067680 is highly expressed during the ostenogenic differentiation of hBMSCs. Functional assays indicated that depletion of circ_0067680 impeded the osteogenic differentiation process of hBMSCs.

Competing endogenous RNA (ceRNA) network plays a significant role in human diseases, ostenogenic differentiation is also included [19, 20]. Through subcellular fractionation and FISH assays, circ_0067680 was found mainly located in the cytoplasm of hBMSCs which indicated that circ_0067680 might be involved in post-transcriptional events by sequestering miRNAs. MiR-4429 is identified to be an inhibitor in multiple malignant tumors, such as clear cell renal cell carcinoma, gastric cancer, ovarian cancer and so on [21–23]. Through our investigation, we discovered that miR-4429 was sponged by circ_0067680 and had a strong affinity with circ_0067680. Moreover, down-regulation of miR-4429 completely restored the suppressive role of circ_0067680 interference on the osteogenic differentiation of hBMSCs.

Wnt signaling pathway is of great significance in the osteogenic differentiation of mesenchymal stem cells [24]. More importantly, CTNNB1 which encodes β-catenin has been revealed to be targeted by miR-214 and promote the osteoblastic differentiation of periodontal ligament stem cells (25). Consistent with these finding, we discovered that CTNNB1 was targeted by miR-4429. The decrease on CTNNB1 expression caused by circ_0067680 knockdown was rescued by miR-4429 inhibition. Rescue assays attested that up-regulation of CTNNB1 completely countervailed the inhibited osteogenic differentiation of hBMSCs imposed by circ_0067680 silencing. A graphical abstract has been provided for reference.

## Conclusions

In a word, it is determined that circ_0067680 was overexpressed in hBMSCs during the osteogenic differentiation. Moreover, knockdown of circ_0067680 abrogated the osteogenic differentiation of hBMSCs. Furthermore, circ_0067680 interacted with miR-4429 and modulated CTNNB1 expression, thereby participating in the progression of the osteogenic differentiation of hBMSCs via activation of Wnt/β-signaling pathway. Thus we could conclude that circ_0067680 played the promoting role in the osteogenic differentiation of hBMSCs. What is more valuable is that circ_0067680 might be used as an effective biomarker and provide novel insights for exploring therapy strategies of bone diseases.

## Methods

### Cell culture

The hBMSCs and HEK-293T cell were obtained from American Type Culture Collection (ATCC; Manassas, VA, USA). HEK-293T cell was preserved in Dulbecco’s Modified Eagle’s Medium (DMEM) containing 10% FBS (Gibco) while hBMSCs were maintained in Mesenchymal Stem Cell Basal Medium containing 7% FBS. All mediums were maintained under 37 °C with 5% CO_2_.

### Plasmid transfection

Genechem (Shanghai, China) synthesized the shRNAs against circ_0067680 and negative control sh-NC. Besides, pcDNA3.1 vectors were sub-cloned with CTNNB1 and provided by Genechem. MiR-4429 mimics/inhibitor and negative control were provided by RiboBio (Shanghai, China). Lipofectamine 3000 (Invitrogen) was used for cell transfection. The sequences used have been provided in Additional file 3: Table S1.

### Quantitative real-time PCR (RT-qPCR) analysis

Three experiments were implemented. Firstly, total RNA was obtained using TRIzol Reagent (Introgen, Carlsbad, CA, USA), and then reversely transcribed into cDNA using RevertAid First Strand cDNA Synthesis Kit (Thermo fisher, IL, USA). qPCR was performed using SYBR Green PCR Master Mix (Applied Biosystems, Foster City, CA, USA) based on the 2^−ΔΔCt^ method. GAPDH or U6 was internal control. Sequences have been provided in Additional file 3: Table S1.

### Cell counting kit-8 (CCK-8)

Three experiments were implemented. Cells were plated in 96-well plates, and treated with 10 μl of CCK-8 solution (Dojindo, Japan). The absorbance was measured at 450 nm.

### Alkaline phosphatase (ALP) staining

According to the protocol of NBT/BCIP kit (CoWin Biotech, Beijing, China), ALP staining analysis was carried out. In brief, hBMSCs cells were cultured in OM for 7 days. After fixation and staining, cells were treated with ALP Activity Kit. The experiment was independently conducted in triplicate.

### Subcellular fractionation

Three experiments were implemented. Via PARIS™ Kit (Ambion, Austin, TX), cytoplasmic and nuclear elements were separated. GAPDH or U6 was respectively regarded as cytoplasmic and nuclear controls.

### Fluorescent in situ hybridization (FISH)

hBMSCs cells were permeabilized and fixed, and then hybridized with the RNA FISH probe mix for circ_0067680 in buffer which was synthesized by RiboBio. Hoechst solution was used to dye nuclei. The experiment was independently conducted in triplicate.

### RNA pull down assay

Three experiments were implemented. The biotinylated circ_0067680 probe or miR-4429 probe was used to treat hBMSCs. Magnetic beads were then added into cells. After collection and purification, RT-qPCR analysis was implemented.

### RNA immunoprecipitation (RIP)

Three experiments were implemented via a Z-Magna RIPTM RNA-binding Protein Immunoprecipitation kit (Millipore Corporation, USA). Cell lysates were treated with Anti-Ago2 (Abcam) antibody and anti-IgG (Abcam) antibody, and analyzed by RT-qPCR.

### Luciferase reporter assay

Three experiments were implemented. The sequence of circ_0067680 or CTNNB1 mRNA 3’-untranslated region (3’-UTR) containing wild-type (Wt) and mutant type (Mut) of miR-4429 was inserted into pmirGLO dual-luciferase vector to form pmirGLO-circ_0067680 Wt/Mut and pmirGLO-CTNNB1 3’-UTR-Wt/Mut respectively. Later, miR-4429 mimics and control mimics were separately co-transfected with the reporter gene into hBMSCs cells. After 48 h, the Dual-Luciferase Reporter Gene Assay Kit (Yeasen, Shanghai, China) was applied to measure luciferase activity.

### TOP/FOP flash assay

After co-transfection with sh‐NC or sh-circ_0067680#1/2, Wnt/β-catenin report vector TOP/FOP was co-transfected into hBMSCs cells. Relative luciferase activities were detected with a dual-luciferase reporter assay Kit (Promega, Madison, WI, USA). The assay was independently carried out in triplicate.

### Western blot analysis

Three experiments were implemented. Separated protein samples were transferred to PVDF membranes (Millipore, Billerica, MA, USA) which subsequently went through incubation with the following primary antibodies against nuclear β-catenin (Abcam, ab32572), p65 (Abcam, ab32536), p-p65 (Abcam, ab76302), AKT (Abcam, ab8805), p-AKT (Abcam, ab38449), c-myc (Abcam, ab32072), cyclin D1 (Abcam, ab16663), RUNX2 (Abcam, ab192256), OSX (Abcam, ab209484), OCN (Abcam, ab93876), C3orf58 (Invitrogen, PA5-26926), Histone H3 (Abcam, ab1791) and GAPDH (Abcam, ab181602) after being blocked with skimmed milk. Afterwards, the blots were incubated with secondary antibody. At last, Chemiluminescence system (GE Healthcare, Chicago, USA) was applied to quantify proteins.

### Statistical analysis

Experimental data were analyzed by SPSS 22.0 statistical software and expressed as mean ± standard deviation (SD). The differences were analyzed with the employment of Student’s t-test or ANOVA. All experiments were independently performed in triplicate. Data was significant when P < 0.05.

## Supplementary Information

**Additional file 1. Figure S1** (A) The viability of hBMSCs was detected by CCK-8 assay after induction of osteogenic differentiation at 7 and 14 days. (B) ALP activity of hBMSCs was tested by ALP staining assay after induction of osteogenic differentiation at 7 and 14 days. (C-D) Expression of RUNX2, OSX and OCN was analyzed by RT-qPCR and western blot after induction of osteogenic differentiation at 7 and 14 days. **P < 0.01.

**Additional file 2. Figure S2** (A-B) C3orf58 expression was examined when circ_0067680 was inhibited.

**Additional file 3. Table S1** Sequences of transfected plasmids.

## Data Availability

Not available.

## Abbreviations

circRNAs: Circular RNAs
hBMSCs: Human bone marrow-derived mesenchymal stem cells
miR-4429: MicroRNA-4429
CTNNB1: Catenin beta 1
ATCC: American Type Culture Collection
DMEM: Dulbecco's Modified Eagle's Medium
RT-qPCR: Quantitative real-time PCR
ALP: Alkaline phosphatase
FISH: Fluorescent in situ hybridization
RIP: RNA immunoprecipitation
3′-UTR: 3′-Untranslated region
Wt: Wild-type
Mut: Mutant type
SD: Standard deviation
gDNA: Genomic DNA
Act D: Actinomycin D
ceRNA: Competing endogenous RNA

## References

[1] Hayashi M, Nakashima T, Yoshimura N, Okamoto K, Tanaka S, Takayanagi H (2019). Autoregulation of osteocyte Sema3A orchestrates estrogen action and counteracts bone aging. Cell Metab.

[2] Wang J, Liu S, Li J, Zhao S, Yi Z (2019). Roles for miRNAs in osteogenic differentiation of bone marrow mesenchymal stem cells. Stem Cell Res Ther.

[3] Dong CL, Liu XH, Wu L (2019). Research and development of osteogenic differentiation of bone marrow mesenchymal stem cells. China J Orthopaed Traumatol.

[4] Kassem M, Abdallah BM, Saeed H (2008). Osteoblastic cells: differentiation and trans-differentiation. Arch Biochem Biophys.

[5] Zhang M, Jia L, Zheng Y (2019). circRNA expression profiles in human bone marrow stem cells undergoing osteoblast differentiation. Stem Cell Rev Rep.

[6] Guo Z, Zhao L, Ji S, Long T, Huang Y, Ju R, et al. CircRNA-23525 regulates osteogenic differentiation of adipose-derived mesenchymal stem cells via miR-30a-3p. Cell Tissue Res. 2020. 10.1007/s00441-020-03305-7 33151455

[7] Han S, Kuang M, Sun C, Wang H, Wang D, Liu Q (2020). Circular RNA hsa_circ_0076690 acts as a prognostic biomarker in osteoporosis and regulates osteogenic differentiation of hBMSCs via sponging miR-152. Aging.

[8] Li X, Zheng Y, Zheng Y, Huang Y, Zhang Y, Jia L (2018). Circular RNA CDR1as regulates osteoblastic differentiation of periodontal ligament stem cells via the miR-7/GDF5/SMAD and p38 MAPK signaling pathway. Stem Cell Res Ther.

[9] Wang Y, Zhang X, Shao J, Liu H, Liu X, Luo E (2017). Adiponectin regulates BMSC osteogenic differentiation and osteogenesis through the Wnt/β-catenin pathway. Sci Rep.

[10] Zhang Y, Wang H, Yin T, Liu Y, Zhou W, Fan X, et al. TMEM18 inhibits osteogenic differentiation of rat bone marrow-derived mesenchymal stem cells by inactivating β-catenin. Exp Cell Res. 2019;383(1):111491. 10.1016/j.yexcr.2019.07.004 31288024

[11] Zhou J, Zhang S, Chen Z, He Z, Xu Y, Li Z (2019). CircRNA-ENO1 promoted glycolysis and tumor progression in lung adenocarcinoma through upregulating its host gene ENO1. Cell Death Dis.

[12] Shen GY, Ren H, Huang JJ, Zhang ZD, Zhao WH, Yu X (2018). Plastrum testudinis extracts promote BMSC proliferation and osteogenic differentiation by regulating Let-7f-5p and the TNFR2/PI3K/AKT signaling pathway. Cell Physiol Biochem.

[13] An J, Li G, Zhang J, Zhou H, Jiang J, Wang X (2019). GNAS knockdown suppresses osteogenic differentiation of mesenchymal stem cells via activation of Hippo signaling pathway. J Cell Physiol.

[14] Fang Y, Xue Z, Zhao L, Yang X, Yang Y, Zhou X (2019). Calycosin stimulates the osteogenic differentiation of rat calvarial osteoblasts by activating the IGF1R/PI3K/Akt signaling pathway. Cell Biol Int.

[15] Bian Y, Xiang J. Salvianolic acid B promotes the osteogenic differentiation of human periodontal ligament cells through Wnt/β-catenin signaling pathway. Arch Oral Biol. 2020;113:104693. 10.1016/j.archoralbio.2020.104693 32179247

[16] Huang X, Cen X, Zhang B, Liao Y, Zhu G, Liu J (2019). Prospect of circular RNA in osteogenesis: a novel orchestrator of signaling pathways. J Cell Physiol.

[17] Beigi F, Schmeckpeper J, Pow-Anpongkul P, Payne JA, Zhang L, Zhang Z (2013). C3orf58, a novel paracrine protein, stimulates cardiomyocyte cell-cycle progression through the PI3K-AKT-CDK7 pathway. Circ Res.

[18] Huang G, Kang Y, Huang Z, Zhang Z, Meng F, Chen W (2017). Identification and characterization of long non-coding RNAs in osteogenic differentiation of human adipose-derived stem cells. Cell Physiol Biochem.

[19] Gu X, Li M, Jin Y, Liu D, Wei F (2017). Identification and integrated analysis of differentially expressed lncRNAs and circRNAs reveal the potential ceRNA networks during PDLSC osteogenic differentiation. BMC Genet.

[20] Smillie CL, Sirey T, Ponting CP (2018). Complexities of post-transcriptional regulation and the modeling of ceRNA crosstalk. Crit Rev Biochem Mol Biol.

[21] Pan H, Hong Y, Yu B, Li L, Zhang X (2019). miR-4429 inhibits tumor progression and epithelial–mesenchymal transition via targeting CDK6 in clear cell renal cell carcinoma. Cancer Biother Radiopharm.

[22] He H, Wu W, Sun Z, Chai L (2019). MiR-4429 prevented gastric cancer progression through targeting METTL3 to inhibit m(6)A-caused stabilization of SEC62. Biochem Biophys Res Commun.

[23] Zhu YM, Chen P, Shi L, Zhu T, Chen X (2020). MiR-4429 suppresses the malignant development of ovarian cancer by targeting YOD1. Eur Rev Med Pharmacol Sci.

[24] Houschyar KS, Tapking C, Duscher D, Harati K, Wallner C, Wagner JM (2019). Regulation of bone metabolism by the Wnt signaling pathway. Handchirurgie, Mikrochirurgie, plastische Chirurgie : Organ der Deutschsprachigen Arbeitsgemeinschaft fur Handchirurgie : Organ der Deutschsprachigen Arbeitsgemeinschaft fur Mikrochirurgie der Peripheren Nerven und Gefasse.

[25] Cao F, Zhan J, Chen X, Zhang K, Lai R, Feng Z (2017). miR-214 promotes periodontal ligament stem cell osteoblastic differentiation by modulating Wnt/β-catenin signaling. Mol Med Rep.

