# Supplementary figures and images for: Circ_0067680 expedites the osteogenic differentiation of human bone marrow-derived mesenchymal stem cells through miR-4429/CTNNB1/Wnt/β-catenin pathway
Source: Biol Direct. 2021 Oct 14;16:16. doi: 10.1186/s13062-021-00302-w (PMC8515698; doi:10.1186/s13062-021-00302-w)

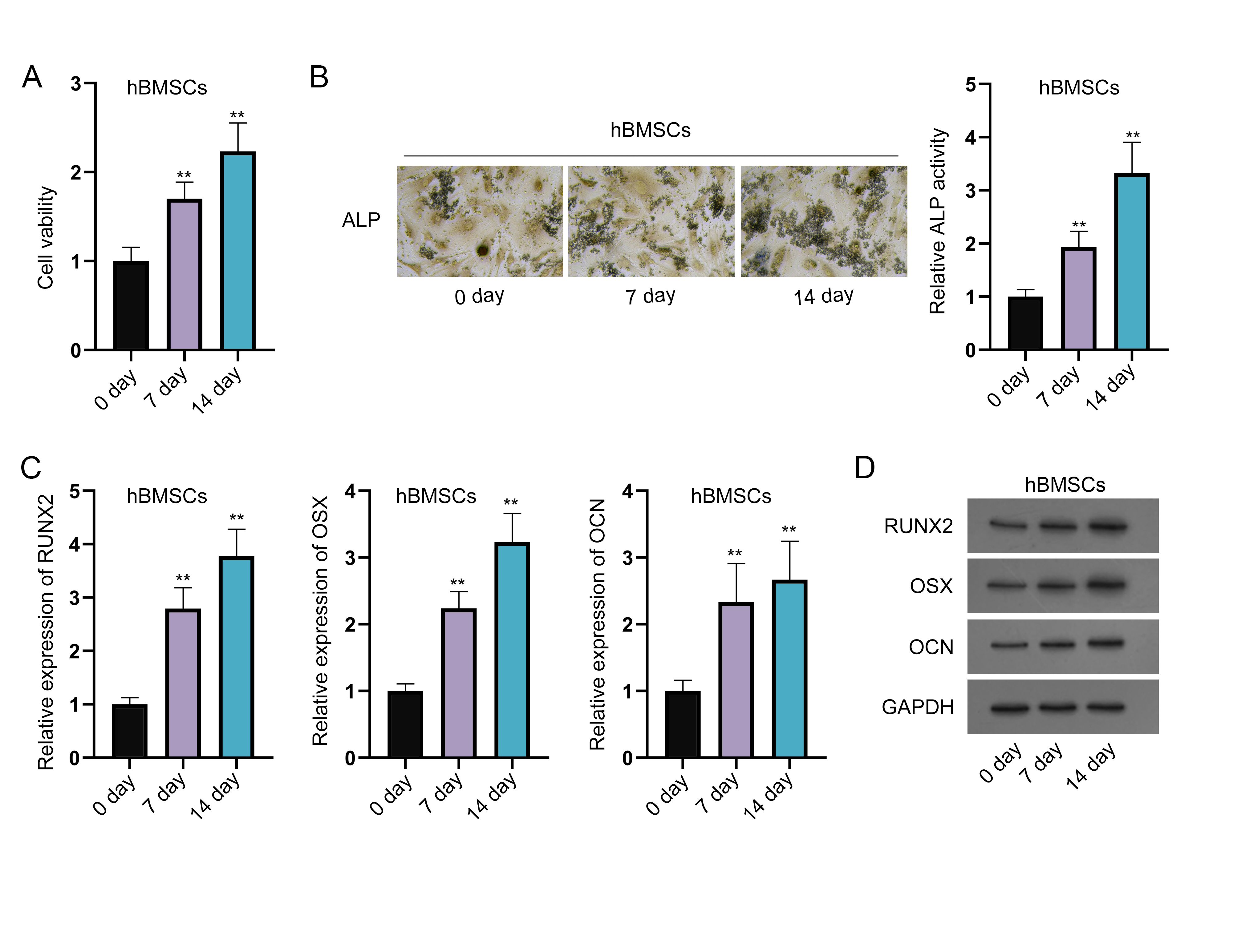

Supplement: Supplementary file 1 — Additional file 1. Figure S1 (A) The viability of hBMSCs was detected by CCK-8 assay after induction of osteogenic differentiation at 7 and 14 days. (B) ALP activity of hBMSCs was tested by ALP staining assay after induction of osteogenic differentiation at 7 and 14 days. (C-D) Expression of RUNX2, OSX and OCN was analyzed by RT-qPCR and western blot after induction of osteogenic differentiation at 7 and 14 days. **P < 0.01. [file 13062_2021_302_MOESM1_ESM.tif]

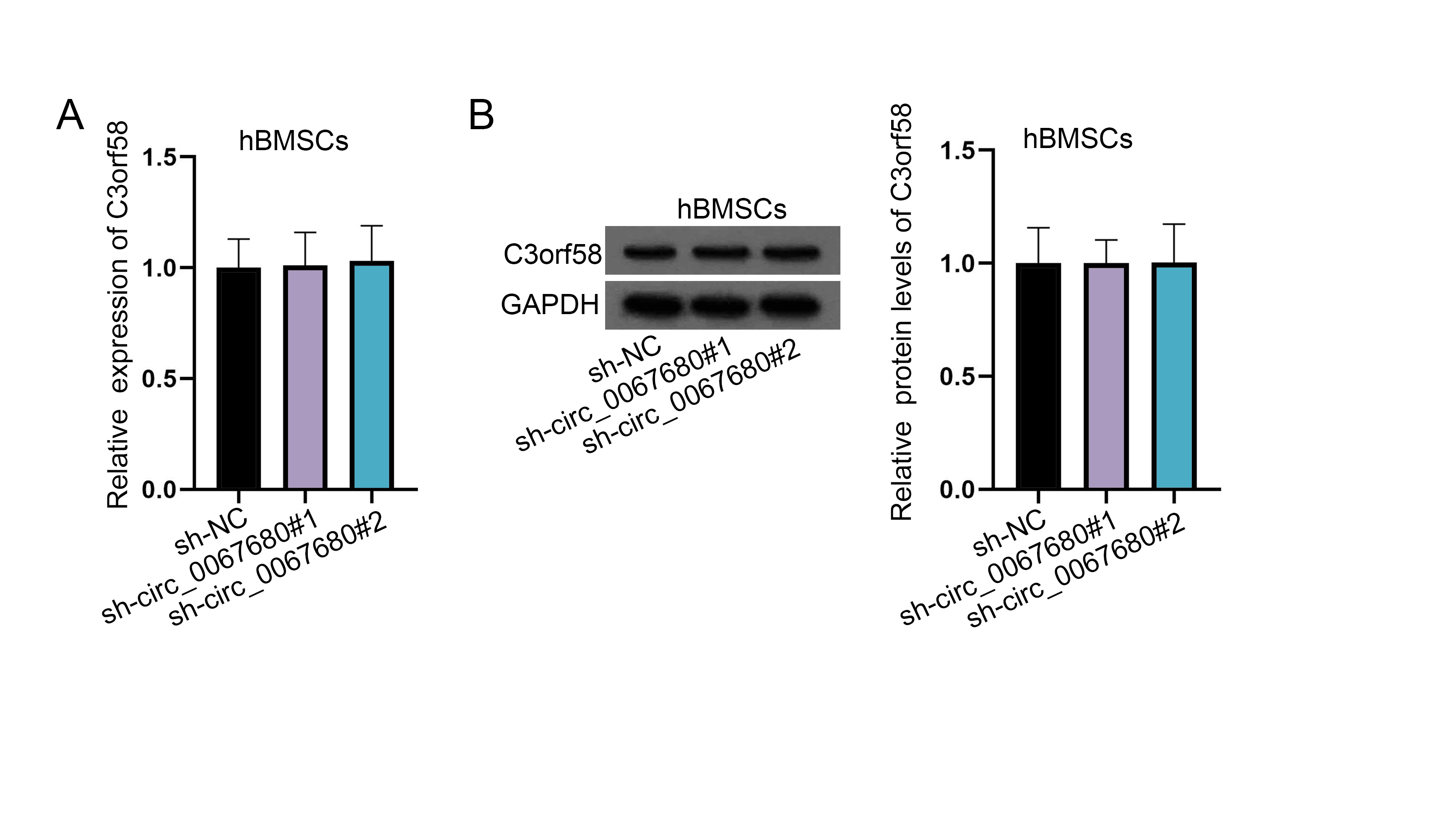

Supplement: Supplementary file 2 — Additional file 2. Figure S2 (A-B) C3orf58 expression was examined when circ_0067680 was inhibited. [file 13062_2021_302_MOESM2_ESM.tif]
